# Paeoniflorin Alleviates Skeletal Muscle Atrophy in Ovariectomized Mice through the ERα/NRF1 Mitochondrial Biogenesis Pathway

**DOI:** 10.3390/ph15040390

**Published:** 2022-03-23

**Authors:** Ki-Sun Park, Hyungjun Kim, Hye Jin Kim, Kang-In Lee, Seo-Young Lee, Jieun Kim

**Affiliations:** 1 KM Science Research Division, Korea Institute of Oriental Medicine, Daejeon 34054, Korea; heyjoon73@kiom.re.kr (H.K.); popigletoh@kiom.re.kr (K.-I.L.); 09seoyoung03@kiom.re.kr (S.-Y.L.); jieunkim@kiom.re.kr (J.K.); 2 KM Convergence Research Division, Korea Institute of Oriental Medicine, Daejeon 34054, Korea; kimhyejin@kiom.re.kr

**Keywords:** muscle atrophy, post-menopausal women, estrogen insufficiency, paeoniflorin, mitochondrial biogenesis, anti-inflammation, muscle differentiation

## Abstract

Muscle atrophy in postmenopausal women is caused by estrogen deficiency and a variety of inflammatory factors, including tumor necrosis factor alpha (TNFα). Paeoniflorin (PNF), a natural compound with anti-inflammatory properties, improves estradiol synthesis. Here, we demonstrate that PNF inhibits the progression of TNFα-induced skeletal muscle atrophy after menopause by restoring mitochondrial biosynthesis. Differentiated myoblasts damaged by TNFα were restored by PNF, as evident by the increase in the expression of myogenin (MyoG) and myosin heavy chain 3 (Myh3)—the markers of muscle differentiation. Moreover, diameter of atrophied myotubes was restored by PNF treatment. TNFα-repressed nuclear respiratory factor 1 (NRF1) and mitochondrial transcription factor A (TFAM) (a major regulator of mitochondrial biosynthesis) were restored by PNF, via regulation by estrogen receptor alpha (ERα), an upregulator of NRF1. This mechanism was confirmed in ovariectomized (OVX) mice with a ~40% reduction in the cross-sectional area of the anterior tibialis muscle. OVX mice administered PNF (100, 300 mg/kg/day) for 12 weeks recovered more than ~20%. Behavioral, rotarod, and inverted screen tests showed that PNF enhances reduced muscle function in OVX mice. ERα restored expression of mitofusin 1 (MFN1) and mitofusin 2 (MFN2) (mitochondrial fusion markers) and dynamin-related protein (DRP1) and fission 1 (FIS1) (mitochondrial fission markers). Therefore, PNF can prevent muscle atrophy in postmenopausal women by inhibiting dysfunctional mitochondrial biogenesis.

## 1. Introduction

Skeletal muscle, which accounts for approximately 40% of our body weight, plays a key role in converting chemical energy into mechanical energy to generate force, control posture, and creates the mobility for action [1,2]. Muscle atrophy and muscle wasting under physiological or pathological conditions lead to poor quality of life and are associated with various diseases [3]. Aging is an unavoidable physiological phenomenon of muscle atrophy; furthermore, muscle atrophy is more pronounced in postmenopausal women than in men [4,5]. Reduction in estrogen levels is the first factor responsible for muscle atrophy in menopausal women [6,7,8]. A prolonged decrease in estrogen levels impairs muscle differentiation, regeneration, and contraction, causing aging and eventually muscle loss and atrophy. Indeed, injection of 17β-estradiol (E2) in OVX, estrogen-deficient animal models can restore damaged mitochondrial biogenesis and the decreased cross-sectional area (CSA) of the tibialis anterior (TA) muscle [8,9,10]. The second factor causing muscle atrophy is the excessive secretion of various inflammatory factors [10]. Postmenopausal women overproduce cytokines, such as TNFα and interleukins (IL1β and IL6) [11,12,13]. The correlation between TNFα and muscle atrophy has been well studied. Mechanistically, TNFα induces the generation of reactive oxygen species in the mitochondria and activates nuclear factor kappa-light-chain-enhancer of activated B cells (NF-κB), signaling the breakdown of proteins in mature muscles, and finally, promotes muscle catabolism through the ubiquitin/proteasome pathway [11,14,15]. Therefore, to prevent and treat aging-induced muscle atrophy, including that in postmenopausal women, new anti-inflammatory substances that can restore the function of mitochondria should be developed.

PNF is a well-known bioactive compound in the extracts of natural herbs, such as *Paeonia lactiflora* and *P. suffruticosa* [16,17]. In view of the remarkable anti-inflammatory properties of PNF, numerous studies have been conducted on its effect in improving inflammatory conditions, such as rheumatoid arthritis, inflammatory bowel disease, psoriasis, and sepsis [18,19]. PNF inhibits the secretion of cytokines, such as TNFα, IL1β, and IL6, and exhibits therapeutic effects through the NF-κB pathway [20,21]. Although not acting as an anti-inflammatory agent, PNF induces the degradation of mutant androgen receptor (AR) to eliminate skeletal muscle atrophy in patients with spinal bulbar atrophy (SBMA) [22]. In addition, PNF can help improve menopause because it promotes estradiol synthesis by increasing the activity of aromatase, which converts testosterone to estrogen [23,24,25]. Therefore, PNF has the potential as a therapeutically active compound that simultaneously alleviates diseases caused by hormonal imbalance as well as inflammatory diseases to prevent skeletal muscle atrophy.

The effects of PNF on muscle atrophy are unclear; furthermore, there have been no studies focusing on the mechanisms to alleviate postmenopausal muscle atrophy using the OVX animal model. In this study, we aimed to identify the role of PNF in restoring mitochondrial biogenesis in muscle atrophy caused by TNFα.

## 2. Results

### 2.1. PNF Restores Atrophied Muscle by Inhibiting Apoptotic Signals Induced by TNFα

**Figure 1:**
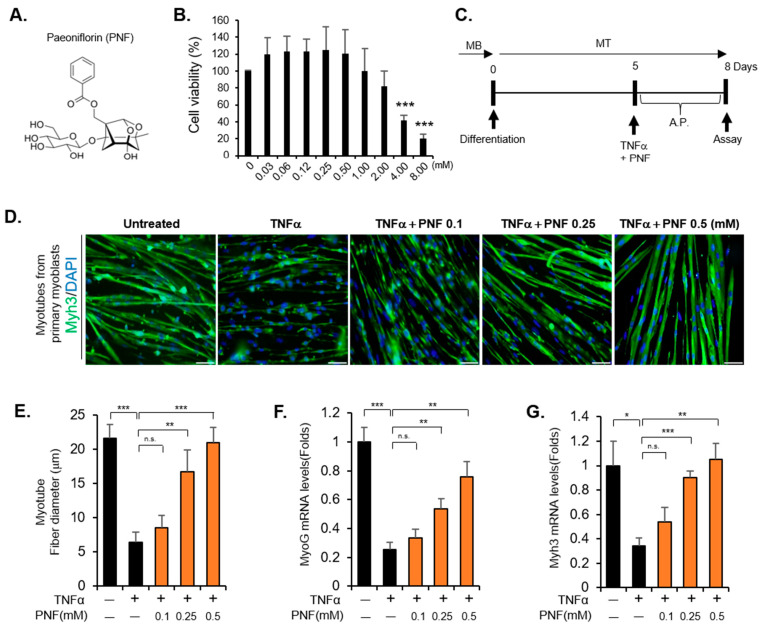
PNF improves TNFα-induced muscle atrophy in primary differentiated myoblasts and C2C12 cells: (**A**) Chemical structure of PNF. (**B**) Effects of PNF on the viability of differentiated C2C12 cells treated with the indicated concentrations for 72 h. (*n* = 3). (**C**) Schematic representation of the in vitro experimental protocol with atrophied muscle. (**D**) Primary differentiated muscles were stained with Myh3. DAPI indicates the nucleus. The concentration of TNFα used was 10 ng/mL, and that of PNF was 0.1, 0.25, and 0.5 mM. Scale bar: 100 µm. (**E**) Quantitation of 200-myotube fiber diameter in primary differentiated myoblasts. Fiber diameter was measured using the Image J program. (**F**,**G**) Expression levels of markers for differentiated muscle, MyoG and Myh3, in differentiated C2C12 cells. (*n* = 3) * *p* < 0.05, ** *p* < 0.01, and *** *p* < 0.001. N.S., not significant.

To determine the cytotoxicity of PNF, cell viability was measured in primary differentiated myoblasts and differentiated C2C12 cells. No toxicity was observed up to 1.0 mM, whereas at concentrations above 2 mM, the survival was reduced. At 4 mM, significant toxicity was observed (Figure 1A,B, Supplementary Figure S1). Subsequently, for the experiment to assess the recovery of muscle atrophy by PNF treatment, primary myoblasts were differentiated for 5 days to obtain myotubes. Thereafter, the cells were treated with TNFα (10 ng/mL) for 3 days to induce muscle atrophy (Figure 1C). The myotube diameter was measured by staining with Myh3—a representative marker of myoblast differentiation. The diameter of the atrophied muscle was reduced by approximately 70% by TNFα. However, PNF pretreatment was able to rescue myotubes from TNFα-induced muscle impairment in a dose-dependent manner (Figure 1D,E). Identical results were obtained for mRNA levels of muscle differentiation markers, MyoG and Myh3. These markers were upregulated by PNF, which is consistent with the results on muscle fiber diameter (Figure 1F,G). Therefore, PNF appears to have an inhibitory effect on the inflammatory factor, TNFα, in muscle atrophy.

**Figure 2:**
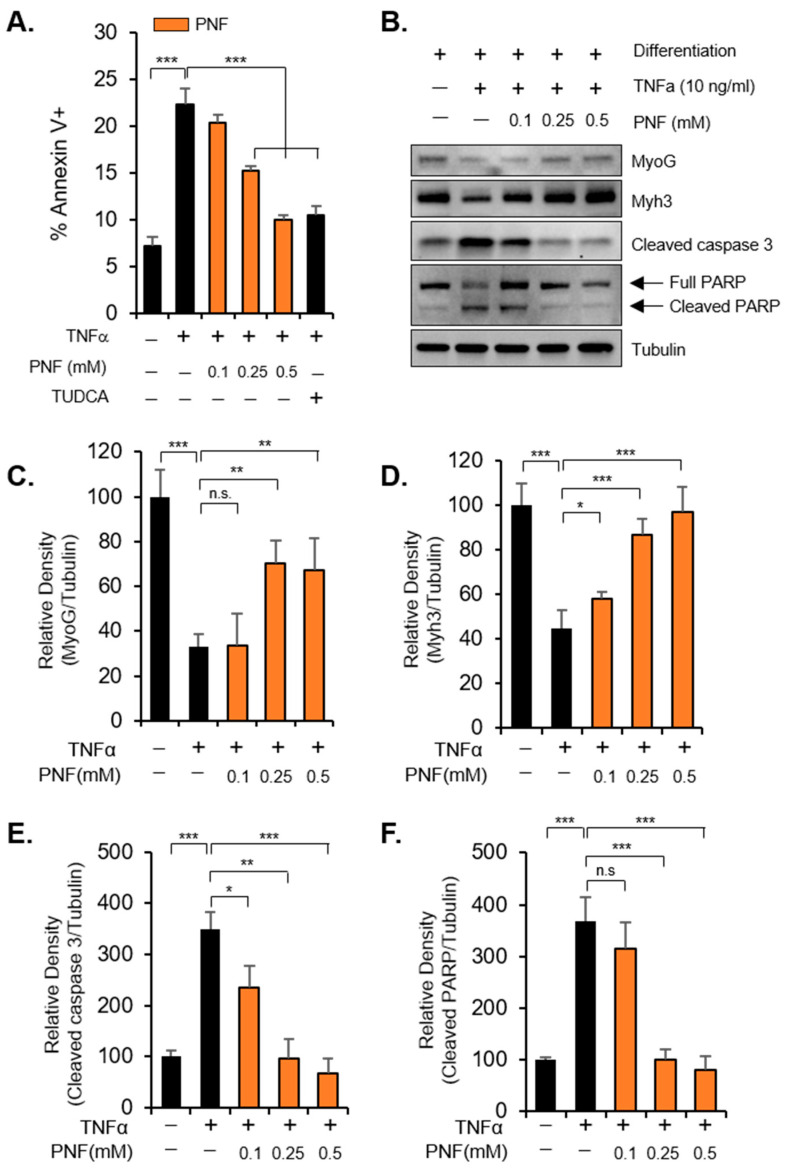
PNF recovers apoptotic signal in C2C12 cells: (**A**) Analysis of annexin V staining in TNFα- and PNF-treated C2C12 cells. The concentration of TNFα used was 10 ng/mL, and that of PNF was 0.1, 0.25, and 0.5 mM (*n* = 3). (**B**) Immunoblotting was performed using protein extract from differentiated muscle. Tubulin was used as an internal control (*n* = 3). (**C**–**F**) Relative protein levels, as determined using densitometry of protein bands in Western blot analysis, from three independent experiments, after normalization to tubulin. Results are represented as the relative percentage (*n* = 3). * *p* < 0.05, ** *p* < 0.01, and *** *p* < 0.001. N.S., not significant.

TNFα-induced atrophy of muscle depends on apoptotic signals. Therefore, to confirm whether PNF can control apoptotic signals, Annexin V staining was performed using flow cytometry. The apoptosis increased by TNFα treatment was reduced by PNF in a dose-dependent manner, indicating that the efficacy of PNF is similar to that of tauroursodeoxycholic acid (TUDCA), a positive control used as an apoptosis inhibitor (Figure 2A). Cleaved caspase 3 and cleaved PARP are representative intracellular apoptosis markers. As expected, the levels of these markers, increased by TNFα treatment, were reduced by PNF in a concentration-dependent manner. Moreover, in accordance with the MyoG and Myh3 mRNA levels, the protein levels were also recovered (Figure 2B–F). Taken together, these data suggest that muscle atrophy induced by TNFα at a high concentration could be improved through mechanisms that inhibit apoptotic signals by PNF treatment.

### 2.2. PNF Increases Mitochondrial Function by Regulating ERα and NRF1

**Figure 3:**
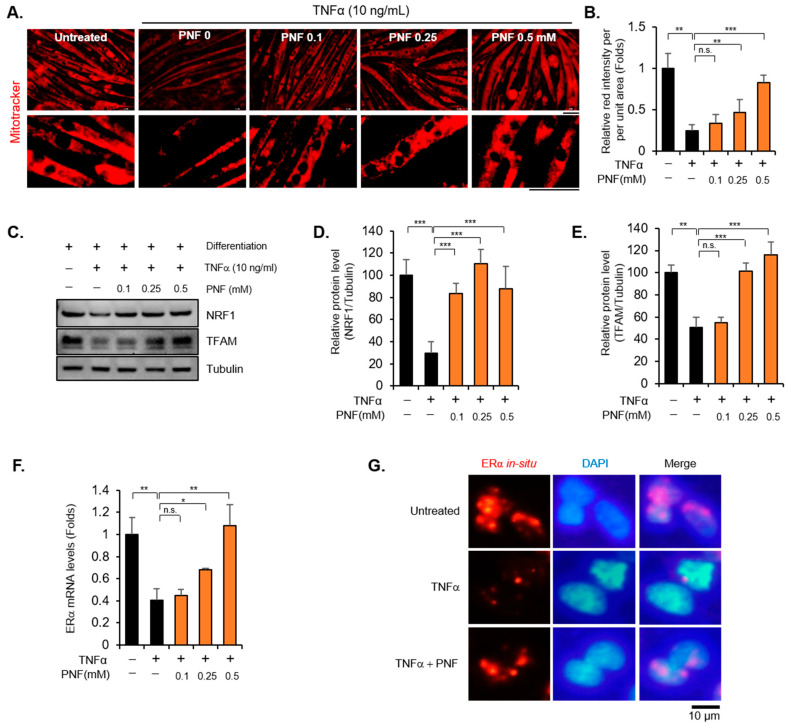
PNF restores mitochondrial biogenesis through NRF1, TFAM, and ERα in C2C12 cells: (**A**) Differentiated myoblasts were stained with MitoTracker. MitoTracker is shown in red. The scale bar indicates 50 µm (*n* = 5). (**B**) Intensity of MitoTracker was quantified in individual muscle cells (*n* = 20 for each group). (**C**) Immunoblotting was performed on proteins extracted from differentiated muscle protein. Tubulin was used as an internal control. (**D**,**E**) Relative densitometric quantification of NRF1 and TFAM from three independent experiments after normalization to tubulin. Results are represented as the relative percentage levels. (**F**) Expression levels of ERα induced by TNFα (10 ng/mL) and PNF in C2C12 cells. (**G**) RNA in situ hybridization staining for ERα (red) and DAPI (blue) in C2C12 cells (*n* = 5) * *p* < 0.05, ** *p* < 0.01, and *** *p* < 0.001. N.S., not significant.

Mitochondria are critical organelles that regulate the homeostasis of muscle metabolism [26]. Mitochondrial dysfunction causes sarcopenia and muscle atrophy, accompanied by apoptotic signals [27,28,29]. Thus, restoring the mitochondrial function of muscle cells can be a good strategy to prevent and treat related diseases, including aging, that cause muscle wasting. We stained mitochondria using mitoTracker Red CMXRox to check whether PNF could restore the function of mitochondria. After treatment with TNFα (10 ng/mL), the fluorescent intensity decreased by more than 70%, whereas in the group treated with PNF, fluorescent intensity increased in a dose-dependent manner (Figure 3A,B). Furthermore, the mRNA levels of peroxisome proliferator-activated receptor co-activator-1α (PGC1α), NRF1, and TFAM, which are master regulators of mitochondrial biogenesis [30], were measured to determine whether PNF recovers mitochondrial biogenesis impaired by TNFα (Supplementary Figure S2). Interestingly, all the markers were mainly reduced by TNFα, but only NRF1 and TFAM were dramatically recovered in a concentration-dependent manner by PNF. These results were also confirmed at the protein level (Figure 3C–E). Because TFAM is known as the target of NRF1 [31,32], we attempted to find the expression of a factor that selectively regulates NRF1. NRF1 is known to be regulated by ERα as an important mediator for mitochondrial unfolded protein response [33,34]. Therefore, the expression of ERα was investigated to understand whether PNF selectively controls ERα. Interestingly, the levels of ERα, repressed by TNFα (10 ng/mL), were recovered; these results are consistent with the expression of NRF1 and TFAM (Figure 3F). To confirm these results, in situ RNA hybridization of ERα was performed and verified using the fluorescence intensity (Figure 3G). As expected, ERα fluorescence was consistent with the RT-PCR results. Taken together, PNF can improve muscle atrophy to recover mitochondrial biogenesis by regulating the expression of NRF1, TFAM, and ERα.

### 2.3. PNF May Improve Muscle Function by Suppressing Muscle Atrophy of OVX

**Figure 4:**
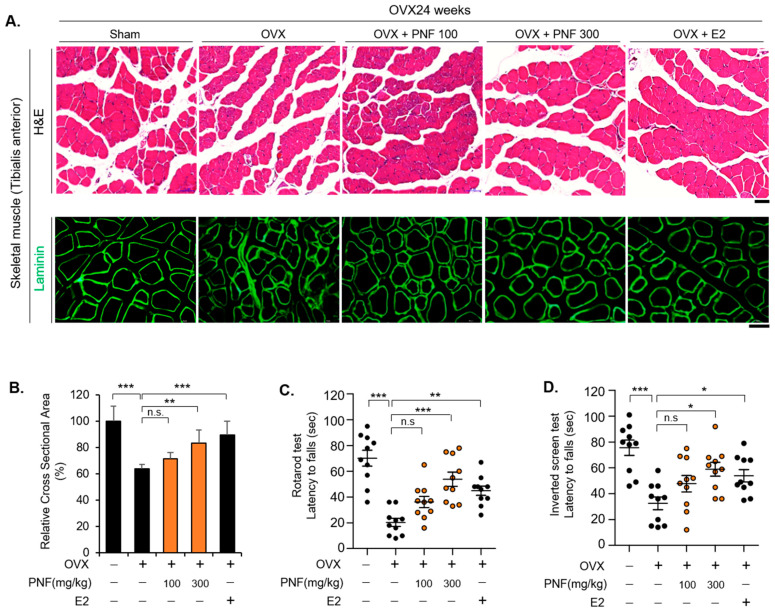
Decreased CSA rescued by PNF in TA muscle of OVX mice: (**A**) Representative hematoxylin and eosin (H&E) and laminin immunofluorescence staining of TA muscle sections. PNF was tested at 100 and 300 mg/kg/day on OVX mice. E2 was used a positive control. Morphology of representative sections visualized using H&E staining. The laminin sections were used for the measurement of fiber area. *n* = 10 for each group. Magnification ×40, Scale bar, 50 µm. (**B**) CSA values were quantified in individual fibers. *n* = 100 for each group. (**C**) Rotarod test of sham and OVX mice administered PNF and E2. *n* = 10. (**D**) Inverted screen test results for sham and OVX mice administered PNF and E2. *n* = 10. * *p* < 0.05, ** *p* < 0.01, and *** *p* < 0.001. N.S., not significant.

The TA muscle of OVX mice was used as a muscle atrophy model after menopause because the muscle fiber diameter and muscle function were reduced [8]. We administered PNF for 12 weeks after removal of the ovaries and administered E2 as a positive control for 4 weeks to check whether PNF recovers muscle loss in OVX mice. To measure the CSA of individual myotubes and fibers, we obtained cryosections and stained them for laminin, a major component localized at the basement membrane of muscle. As expected, the CSA of TA muscle in OVX mice was reduced by approximately 40%. Surprisingly, in the PNF-treated group, no significant changes were observed at a low concentration of 100 mg/kg, but in the group administered a relative higher concentration (300 mg/kg) of PNF, the CSA of the TA muscle was significantly increased. The CSA was also increased in the E2 group (positive control) (Figure 4A,B). To determine whether an increase in CSA can improve the function of mouse muscle, the rotarod and inverted screen tests were performed. The distribution of normality between each group was analyzed. The data in the rotarod (F(4, 45) = 15.892, *p* < 0.001) and inverted screen (F(4, 45) = 8.270, *p* < 0.001) tests showed a normal distribution (Table S3). Mean differences and significance between groups were analyzed using Dunnett’s test (Table S4). Both the tests showed a significant difference at 300 mg/kg of PNF, and it was confirmed that muscle function was restored in the E2 group. These results are consistent with the efficacy of PNF inferred from the analysis of muscle CSA. Therefore, PNF could be used as a potential drug to improve muscle atrophy under conditions of estrogen insufficiency.

### 2.4. Reduced ERα in OVX May Be Maintained by PNF

**Figure 5:**
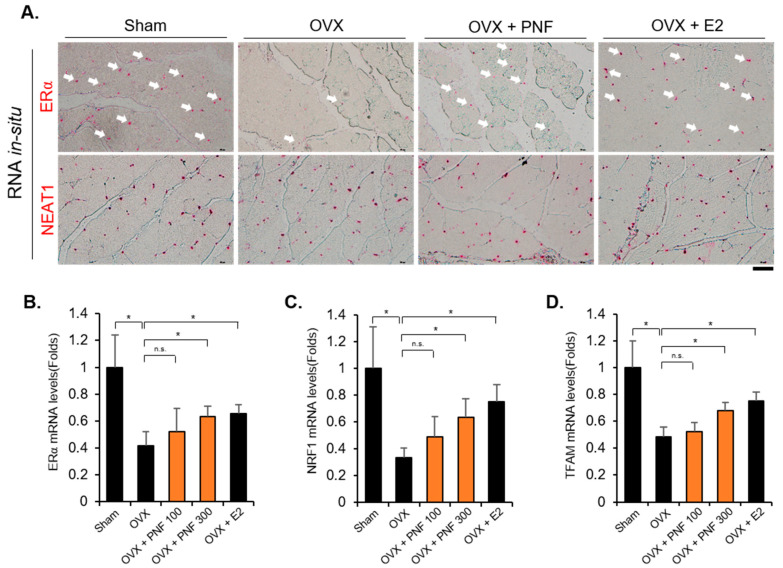
Suppressed ERα, NRF1, and TFAM levels are rescued by PNF in TA muscle of OVX mice: (**A**) RNA in situ hybridization staining for ERα and Neat1 (red) in TA muscle (*n* = 3). Neat1 was used as an internal control. Magnification ×40, Scale bar, 50 µm. (**B**) Representative ERα mRNA in situ hybridization signals from TA muscle. PNF was tested at 300 mg/kg/day on OVX mice. E2 was used as a positive control. (**C**,**D**) Expression of mRNA levels of NRF1 and TFAM in TA muscle. *n* = 3. * *p* < 0.05. N.S., not significant.

OVX not only reduces estrogen levels but also decreases the expression of ERα [35,36]. Because a reduction in ERα levels by TNFα was recovered by PNF, as observed in the in vitro studies, we investigated whether ERα levels were also increased by PNF in OVX mice. As expected, the mRNA levels of ERα were reduced by OVX. However, ERα levels were increased by PNF, and these results were confirmed by ERα RNA in situ hybridization in TA muscle (Figure 5A,B). Neat1, a long-noncoding RNA, was used as an internal control. Neat1 is widely expressed in many tissues, including skeletal muscle [37]. The expression levels of NRF1 and TFAM, which are target genes of ERα, were rescued (Figure 5C,D). These results show that PNF can be used as a new molecule to regulate the mitochondrial biogenesis in OVX mice.

### 2.5. PNF Increases the Mitochondrial Fusion and Fission Markers of OVX

**Figure 6:**
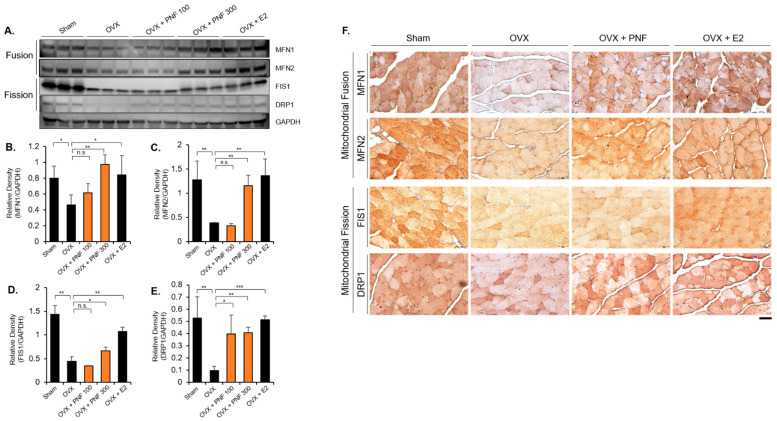
Dysregulated mitochondrial fission and fusion markers are recovered by PNF in TA muscle of OVX mice: (**A**) Immunoblotting was performed using proteins extracted from TA muscle. GAPDH was used as an internal control. *n* = 3. (**B**–**E**) Relative densitometric quantification of MNF1, MFN2, FIS1, and DRP1 levels from three independent experiments after normalization to GAPDH. Results are represented as relative percentages (*n* = 3). (**F**) Representative immunohistochemical staining of MFN1, MFN2, FIS1, and DRP1 in TA muscle. PNF was tested at 300 mg/kg/day on OVX mice. E2 was used a positive control. MFN1 and MFN2 indicate markers of mitochondrial fusion. FIS1 and DRP1 indicate markers of mitochondrial fission. *n* = 5 for each group. Magnification ×40, Scale bar, 50 µm. * *p* < 0.05, ** *p* < 0.01, and *** *p* < 0.001. N.S., not significant.

Mitochondrial fusion and fission are required to maintain mitochondrial biogenesis for adapting to new environmental conditions [36,38,39]. Disruption of mitochondrial biogenesis leads to aging and various diseases. Interestingly, both mitochondrial fusion and fission functions are decreased in muscle of OVX mice and are restored through E2 hormone replacement [9]. Therefore, protein levels were comparatively analyzed to determine whether PNF regulates the expression of mitochondrial (MFN1 and MFN2) and mitochondrial fission (FIS1 and DRP1) markers. These markers were significantly decreased in the OVX group compared with those in the sham group. Although MFN1, MFN2, and DRP1 levels increased in a dose-dependent manner, FIS1 only significantly increased at high concentrations (300 mg/kg). In the E2 group (positive control), the levels of all the markers were recovered, as expected (Figure 6A–E). Results of immunohistochemistry analysis showed that the levels of the stained markers were reduced in OVX muscle. However, mice administered PNF (300 mg/kg) showed restoration of the levels of mitochondrial biogenesis markers, similar to that in the E2-administered mice (Figure 6F).

## 3. Discussion

In women, muscle loss is promoted earlier than in men [4]. Furthermore, it is suggested that muscle loss occurs around the time of menopause [40]. On an average, postmenopausal women suffer loss of muscle at a rate of 0.6% per year, and the levels of potassium, a marker of lean body, continue to decline for the first 3 years after menopause [4]. In fact, muscle strength and muscle mass of women aged 65–69 years are known to be weaker than those of men aged 85–89 years [41]. Loss of muscle in postmenopausal women is caused by inflammatory factors, such as increased TNFα and IL1β levels, as well as due to estrogen deficiency [42,43]. In this study, we found that PNF, a bioactive compound derived from natural products, restores mitochondrial biogenesis impaired by TNFα to improve muscle atrophy. Furthermore, we found that the reduced CSA of TA in OVX mice was effectively recovered by PNF, resulting in the recovery of muscle strength. Although PNF has a potential therapeutic effect on inflammatory disorders in vitro and in vivo [18], studies on muscle atrophy caused by inflammation have been insufficient. To address these questions, we confirmed that muscle fiber was damaged because of the exposure of differentiated myoblasts to TNFα. However, myotubes treated with PNF showed dramatic recovery of muscle fiber in a dose-dependent manner. In addition, PNF increased the expression of muscle differentiation markers, MyoG and Myh3, in myotubes damaged by TNFα. These results imply that the previously known anti-inflammatory effect of PNF can also be applied to muscle cells and PNF can be used as a potential therapeutic compound for muscle atrophy, with traditional anti-inflammatory disease efficacy.

Although apoptosis is an essential intracellular process to maintain the number of proliferating cells, excessive apoptosis in post mitotic tissues, such as skeletal muscle, is considered a pathological process that causes muscle atrophy [44]. In particular, in the case of aging and denervation-induced atrophy, apoptotic signals are recognized as the final stage in muscle loss and atrophy [45,46]. In this context, previous studies have shown that excessive production of TNFα in various diseases causes muscle satellite cell incompetence, resulting in negative effects on the differentiation of muscle cells. However, even in muscles that are already mature, TNFα induces apoptotic signals and promotes muscle loss [47,48,49]. As mentioned earlier, TNFα, which is excessively expressed after menopause, can promote degeneration of mature muscle proteins. Therefore, activation of apoptotic signals can act as an important mechanism to alleviate muscle atrophy. We investigated whether these mechanisms were inhibited by PNF after muscle cells were exposed to TNFα, which caused apoptosis. First, flow cytometry analysis using Annexin V was performed to identify apoptotic cell death; apoptosis was suppressed depending on the concentration of PNF. At a concentration of 0.5 mM, the effect was similar to that of TUDCA, used as positive control, which shows remarkable protection of myotubes by PNF. TNFα increased the levels of apoptosis marker proteins, cleaved caspase 3 and PARP, by more than three times; however, this increase was inhibited by PNF in a dose-dependent manner. In contrast to the activated apoptotic signal, the levels of differentiation markers, MyoG and Myh3, recovered in a PNF dose dependent manners. Therefore, we suggest that PNF is a target therapeutic bioactive compound that inhibits apoptosis in muscle atrophy.

Even if apoptosis is the final step in muscle loss to induce muscle dysfunction, the mechanism of the upstream signals is complex. From the viewpoint of the mechanical function of mitochondria, mitochondria produce ATP for the survival of cells and are essential for muscle contraction and muscle cell quality control [44,46]. The dysfunction of organelles due to aging and disuse atrophy induces dysregulation of mitochondrial biogenesis, which eventually promotes muscle apoptosis. We investigated the expression of NRF1, TFAM, and PGC1α, the master regulators of mitochondrial biogenesis, to check whether PNF could recover the dysfunctional mitochondrial biogenesis in OVX mice. PNF could restore the reduced expression of NRF1 and TFAM in OVX mice, but PGC1α could not. In addition, we confirmed that the NRF1 and TFAM mRNA levels in OVX mice administered PNF were increased, as well as in positive control mice injected with E2. These results were consistent with those of previous studies in which the expression of NRF1 was increased by the treatment of E2 in vitro [50]. Luena et al. reported that ERα acts as an upstream regulator of NRF1 to mediate the response of mitochondrial unfolded protein [34]. To investigate whether PNF regulates ERα mRNA expression, we performed in situ hybridization of ERα in C2C12 and TA muscle. Surprisingly, recovery of ERα levels by PNF was noted in TNFα-treated cells and in TA muscle of OVX mice; these results are also consistent with the mRNA levels.

We histologically proved that mitochondrial fusion and fission that were disrupted in OVX mice were recovered by PNF treatment. These results indicate that PNF inhibits muscle atrophy by recovering mitochondrial biogenesis through ERα. Mitochondria require a continuous cycle of fusion and fission to maintain functional homeostasis and dynamics. Interestingly, as observed in this study, both fusion and fission functions were decreased in OVX mice [50]. The decreased estrogen levels, as a consequence of ovariectomy, provoke very complex changes, such as in muscle contractile properties, phenotype, metabolism, and force-generating capacity, at once. Due to these enormous physiological and pathological changes, an unusual phenomenon in which mitochondrial fusion and fission decrease can be found concomitantly. Garbriela et al. confirmed that mitochondrial fusion (MFN1 and MFN2) and fission (FIS1 and DRP1) markers, which had been reduced in OVX, were recovered through E2 replacement, suggesting that decreased mitochondrial biogenesis in OVX was due to estrogen deficiency [50]. David et al. also found that MFN2 plays a key role in regulating muscle mitochondrial damage, and its levels are significantly decreased in aged muscle. In addition, expression of DRP1 was reduced as in OVX mice [51]. In a similar context, Justin et al. studied the mitochondrial and intramyocellular lipid structure of skeletal muscle in young people in their 20s as well as in elderly people in their 70s. The results of their study confirmed that the overall mitochondrial function decreases, and the expression of fusion and fission marker genes decreases concomitantly [52]. Therefore, it can be suggested that aging, including menopause, can promote the impairment of mitochondrial function due to the simultaneous occurrence of disturbance in hormonal homeostasis and increase in the levels of inflammatory factors. Although this study is limited to muscle atrophy, further studies on the efficacy of PNF in other diseases are needed.

## 4. Material and Methods

### 4.1. Animals Study Design

All experiments were performed using 9-week-old C57BL/6 female mice. The mice were purchased from Dooyeol Biotech (DB, Seoul, Korea). Fifty mice were randomly assigned to different groups (Control, OVX, OVX + E2 70 µg/kg/day, and OVX + PNF 100 and 300 mg/kg/day) (*n* = 10/group). After 2 weeks, mice were ovariectomized bilaterally to block estrogen secretion. Estradiol (Cat# E2758, Sigma-Aldrich, St. Louis, MO, USA) and PNF (Cat# P0038, Sigma-Aldrich, St. Louis, MO, USA) were dissolved in distilled water at certain concentrations before use. Three months after surgery, oral administration of PNF was started. PNF was orally administered once daily for 12 weeks, and the estrogen group was injected with E2 (70 µg/kg/day) for 4 weeks to recover the effects of estrogen. After a total of 6 months, behavior tests were conducted, and the mice skeletal muscle tissues were isolated. The animal experimental procedures were performed in accordance with the Korea Institute of Oriental Medicine (KIOM-20-079) and the National Institutes of Health guide for the care and use of laboratory animals (NIH publication No. 8023, revised 1978). At the end of the experiment, the mice were anesthetized using tribromoethanol (150 mg/kg body weight), and their muscle tissues were immediately harvested. 

### 4.2. Cell Culture and Reagents

C2C12 cells were purchased from the American Type Culture Collection (Gaithersburg, VA, USA) and cultured in Dulbecco’s modified Eagle’s medium (DMEM; Cat# 11965092, Life Technologies, Waltham, MA, USA) containing 10% fetal bovine serum with 1% penicillin and streptomycin (Cat# 10378016, Thermo Scientific, Waltham MA, USA). Primary skeletal muscle was isolated from the hind limb of p3 to p4 pups. Briefly, the isolated hind limb was minced using 5 U/mL collagenase D (Cat# 11088858001, Sigma-Aldrich, St. Louis, MO, USA) and 4 U/mL dispase (Cat# 4942078001, Sigma-Aldrich, St. Louis, MO, USA). After triturating by gently pipetting with a 5 mL pipette, the mixture was incubated at 37 °C for 20 min until a fine slurry was obtained. The slurry was filtered through a piece of nylon mesh (70 µm pore size). The cells were centrifuged at 1000 rpm for 5 min. The supernatant was removed, and the pellet was suspended in 10 mL of F10 based primary myoblast growth medium (Cat# 12390-035, Thermo Scientific, Waltham, MA, USA), and then transferred into a normal dish to remove fibroblasts. After plating, the cells were resuspended in 10 mL medium and plated on collagen-coated dish (Cat# 354450, Thermo Scientific, Waltham, MA, USA). To differentiate primary myoblasts and C2C12, DMEM containing 2% horse serum (Cat# 16050130, Thermo Scientific, Waltham, MA, USA) with 1% penicillin and streptomycin was used. Primary myoblast isolation was performed as described previously [53,54], and primary cells were grown at 37 °C in 10% CO_2_ in either F10 medium (Cat# 12390-035, Thermo Scientific, Waltham, MA, USA) supplemented with 10% cosmic calf serum (Cat# SH30413.02, Thermo Scientific, Waltham, MA, USA) and 2.5 µg/mL fibroblast growth factor (Cat# 100-18B, Thermo Scientific, Waltham, MA, USA). PNF and TUDCA (Cat# 0266, Sigma-Aldrich, St. Louis, MO, USA) were prepared by dissolving in dimethylsulfoxide (DMSO) (Sigma-Aldrich). TNFα was dissolved in PBS with 0.5% BSA (B6917, Sigma-Aldrich, St. Louis, MO, USA), and PNF was dissolved in DMSO.

### 4.3. Cell-Viability Assay

PNF was dissolved in DMSO and then filtered through a filter with a 0.2 µm pore size. A total of 1 × 10^3^ cells was plated in 96-well plates, and then, different concentrations of PNF were added. After 72 h, the samples were incubated with MTS (Cat# G3582, Promega, Madison, WI, USA) for 1 h. Absorbance was recorded at 490 nm.

### 4.4. Quantitative Real-Time PCR for RNA Samples

The mRNA levels of MyoG, Myh3, NRF1, TFAM, and PGC1α were determined by quantitative reverse transcriptase-PCR analysis. RNA was obtained from three independent biological samples. A total of 1 × 10^5^ cells was plated in 6-well plates, and, then, different concentrations of PNF were added along with TNFα. For RNA isolation, the RNeasy Plus Kit (Cat# 74034, Qiagen, Valencia, CA, USA) was used. Tissue RNA isolation was performed using the Trizol kit (Cat# 12183555, Thermo Scientific, Waltham, MA, USA). cDNAs were synthesized using the RevertAid First Stand cDNA Synthesis Kit (Cat# K1622, Thermo Scientific, Waltham, MA, USA). The primers used in the study are listed in Table S1.

### 4.5. Annexin V Staining

Cells were seeded onto flat-bottomed 6-well plates and treated with PNF and TUDCA. A total of 1 × 10^5^ cells was plated in 6-well plates, and then, different concentrations of PNF were added. Apoptosis induction was quantified by flow cytometry using the Alexa fluor 488 annexin V staining kit (Cat# V13241, Thermo Scientific, Waltham, MA, USA). According to the manufacturer’s protocol, 5 µL annexin V working solution was added to each 100 µL of cell suspension. The cells were incubated for 15 min, and annexin binding buffer was added. The stained cells were analyzed by flow cytometry, and the fluorescence was measured.

### 4.6. Immunofluorescence

Cells were fixed in 4% paraformaldehyde for 10 min, permeabilized with 0.5% Triton X-100 for 10 min, and then, blocked with Sea-block buffer for 1 h (Cat# 37527, Abcam, Cambridge, UK) at 25 °C before incubation with the Myh3 antibody (SantaCruz, Dallas, TX, USA). For Myh3 staining, the antibody was diluted 1:1000 in DAKO diluent buffer (Cat# S080983-2, Agilent, Santa Clara, CA, USA), incubated at 4 °C for 24 h, and visualized using the Alexa-488 secondary antibody from Abcam (Cat# ab150077, Abcam, Cambridge, MA, USA). For nuclear counterstaining, we used 4′,6-diamidino-2-phenylindole (DAPI) (Cat# D1306, Thermo Scientific, Waltham, MA, USA). For mitotracker staining, the probe was diluted in 50 nM DMEM and incubated at 37 °C for 30 min. Mitotracker Red CMXRos kits were obtained from Molecular probes (Cat# M7512, Thermo Scientific, Waltham, MA, USA). For quantification of immunofluorescence, stained myotubes were analyzed using the ImageJ/Fiji open software. The summed pixel intensity was calculated in the area delimited by a contour. To avoid the possible interference in fluorescence by undifferentiated myoblasts, only myotubes with three or more nuclei fused together were selected.

For laminin staining, muscle tissues were frozen in liquid nitrogen and cut in cryostat microtome set at −25 °C. The antibody information is provided in Table S2.

### 4.7. Immunohistochemistry

Immunostaining was performed as previously described with some modifications [55,56]. In brief, deparaffinized slides were heated in Antigen Retrieval Solution (Cat# ab93678, Abcam, Cambridge, MA, USA) for 15 min, and then, were incubated with 3% hydrogen peroxide in methanol for 10 min. To block any non-specific binding protein, the prepared slides were incubated with Sea-block solution (Cat# 37527, Abcam, Cambridge, MA, USA). Primary antibody was diluted in DAKO diluent solution (Cat# S080983-2, Agilent, Santa Clara, CA, USA) and incubated overnight at 4 °C in a humidified chamber. After washing the slides in PBS, they were incubated with secondary antibody (Vector Laboratories, Burlingame, CA, USA) for 1 h at room temperature. The slides were incubated with DAB solution and then counterstained with hematoxylin (Leica, Buffalo Grove, IL, USA). The antibody information is provided in Table S2.

### 4.8. Immunoblotting

A total of 1 × 10^6^ cells was plated in 6-well plates, and, then, different concentrations of PNF were added. Total cell lysates (50 µg) were extracted using mPER buffer (Cat# 78501, Thermo Scientific, Waltham, MA, USA) supplemented with a protease inhibitor cocktail (Cat# 4693116001, Roche, Basel, Swiss). Isolated TA muscles were extracted using tPER buffer (Cat# 75810, Thermo Scientific, Waltham, MA, USA) supplemented with a protease inhibitor cocktail. All antibodies were diluted using the HIKARI signal enhancer solution (Cat# 02267-41, NACALAI, Kyoto, Japan). The antibodies used are listed in Table S2. A Chemidoc MP imaging system was used to produce digital images (Bio-Rad, Hercules, CA, USA). The Image J version 1.52a software was used to quantify the relative protein intensity. The densitometric values of target proteins were normalized to endogenous tubulin and GAPDH levels. 

### 4.9. Rotarod and Inverted Screen Test

The rotarod test was performed as described in a previous study with some modifications [57,58]. Briefly, before the rotarod test, the mice were habituated to stay on the rod for 5 min before the test. In the training session, sham and treated mice were placed on the bar of a rotarod and smoothly accelerated to a maximum of 10 rpm over 5 min. The mice were placed back to the rod after falling, up to three times in one session. The inverted screen test was performed as described previously with some modifications [59]. Briefly, mice were placed on a wire mesh screen in the center. Next, the screen was inverted and held ~50 cm above the soft surface. The time when the mice fell off was recorded.

### 4.10. RNA In Situ Hybridization

The RNA in situ hybridization was performed as described in a previous study with some modifications [60,61]. The ERα single color probe was purchased from Advanced Cell Diagnostics (Cat# 432861, ACD, Newark, CA, USA). In situ RNA hybridization was conducted on paraffin sections using the 2.5 HD red detection kit (Cat# 322360, ACD, Newark, CA, USA). RNA in situ assays were conducted according to the manufacturer’s instructions and were performed as described previously. Briefly, paraffin slides were deparaffinized with xylene and ethanol, and then boiled for 15 min using RNAscope Retrial Regents. Thereafter, the slides were incubated in hydrogen peroxide for 10 min and washed with distilled water. The dried slides were treated with the RNAscope protease plus at 40 °C for 30 min. Target RNA probe exposure was performed in a 40 °C oven for 2 h. To amplify the probe signal, hybridization was performed with AMP (1 to 6) reagents at 40 °C and at 25 °C. 

### 4.11. Statistical Analysis

Statistical analyses were conducted using the GraphPad Prism (Version 9.0) software and one-way analysis of variance (ANOVA) (Prism, San Diego, CA, USA). For the behavior tests, one-way ANOVA was performed using SPSS 21.0 to analyze the difference between OVX and PNF- and E2-treated groups. Data normality was tested using the Kolmogorov–Smirnov and Shapiro–Wilk tests. Dunnett’s multiple comparison test was performed to evaluate enhancement in behaviors, using the group of OVX as a reference. The data were expressed as mean ± standard deviation (SD), and the levels of significance were set at * *p* < 0.05, ** *p* < 0.01, and *** *p* < 0.001.

## 5. Conclusions

In conclusion, we suggest that PNF, a bioactive compound of *P. lactiflora* and *P. suffruticosa*, acts as an anti-inflammatory compound to suppress muscle atrophy by recovering mitochondrial biogenesis. Hormone replacement therapy is suggested as an alternative to prevent muscle atrophy in postmenopausal women, but long-term administration can cause secondary side effects. Therefore, we propose that PNF treatment can act as an alternative to hormone replacement to restore the function of mitochondria and can potentially prevent muscle loss in postmenopausal women.

## Data Availability

Data is contained within the article and Supplementary Materials.

## Supplementary Materials

The following supporting information can be downloaded at: https://www.mdpi.com/article/10.3390/ph15040390/s1, Table S1: Primers for Quantitative PCR (qPCR); Table S2: Antibodies for immunoblot & immunofluorescence; Table S3: Normality distribution (Rotarod test and Inverted screen test); Table S4: Dunnett’s test (Rotarod test and Inverted screen test); Figure S1: Effects of PNF on the viability of primary differentiated myoblast cells treated with the indicated concentrations for 72 h. (*n* = 3). * *p* < 0.05, ** *p* < 0.01, and *** *p* < 0.001; Figure S2: PNF selectively recovers the TNFα-induced reduction of NRF1 and TFAM transcripts, but not PGC1α in C2C12 cells. TNFα (10 ng/mL), PNF was treated in cells for 24 hours. (A) PGC1a, (B) NRF1, (C) TFAM. * *p* < 0.05, ** *p* < 0.01, and *** *p* < 0.001. N.S., not significant.
