# Supplementary material for: Paeoniflorin Alleviates Skeletal Muscle Atrophy in Ovariectomized Mice through the ERα/NRF1 Mitochondrial Biogenesis Pathway
Source: Pharmaceuticals (Basel). 2022 Mar 23;15(4):390. doi: 10.3390/ph15040390 (PMC9025649; doi:10.3390/ph15040390)
Supplement: Supplementary file 1 [file pharmaceuticals-15-00390-s001.zip › pharmaceuticals-1582793-supplementary.pdf]

Supporting Information for

Paeoniflorin alleviates skeletal muscle atrophy in ovariectomized mice through the ERα/NRF1 mitochondrial biogenesis pathway

Ki-Sun Park [1,*], Hyungjun Kim [1], Hye Jin Kime [2], Kang In Lee [1], Seoyoung Lee [1], Jieun Kim [1]

[1] KM Science Research Division, Korea Institute of Oriental Medicine, Daejeon 34054, Korea

[2] KM Convergence Research Division, Korea Institute of Oriental Medicine, Daejeon 34054, Korea

[*] Corresponding: kisunpark@kiom.re.kr

Table S1. Primers for Quantitative PCR (qPCR).

Species	Gene name		Sequence (5'to 3')	Tm (℃)
Mouse	MyoG	F	GAGACATCCCCCTATTTCTACCA	60
		R	GCTCAGTCCGCTCATAGCC	60
Mouse	Myh3	F	GAGGAGGCTGATGAACAAGC	60
		R	TCCGGCTAGAGGTGAAGTCG	60
Mouse	ERα	F	TCTGCCAAGGAGACTCGCTACT	60
		R	GGTGCATTGGTTTGTAGCTGGAC	60
Mouse	NRF1	F	GGCAACAGTAGCCACATTGGCT	60
		R	GTCTGGATGGTCATTTCACCGC	60
Mouse	PGC1α	F	GAATCAAGCCACTACAGACACCG	60
		R	CATCCCTCTTGAGCCTTTCGTG	60
Mouse	TFAM	F	GAGGCAAAGGATGATTCGGCTC	60
		R	CGAATCCTATCATCTTTAGCAAGC	60
Mouse	GAPDH	F	TCAATGAAGGGGTCGTTGAT	60
		R	CGTCCCGTAGACAAAATGGT	60

Table S2. Antibodies for immunoblot & immunofluorescence.

Antibody	Source	Catalog No.
Myh3	Santa Cruz	SC-53091

MyoG	Invitrogen	MA5-11486
Cleaved Caspase 3	Cell signaling	9664
PARP	Cell signaling	9542
Tubulin	Abcam	Ab7291
NRF1	Proteintech	12482-1-AP
TFAM	Proteintech	22586-1-AP
Laminin	Abcam	Ab11575
GAPDH	Cell signaling	2188
MFN1	Proteintech	13798-1-AP
MFN2	Proteintech	12186-1-AP
FIS1	Proteintech	10956-1-AP
DRP1	Abcam	Ab154879

Table S3. Normality distribution (Rotarod test and Inverted screen test).

Tests of Normality

grp		Kolmogorov-Smirnov[a]			Shapiro-Wilk		
		Statistic	df	Sig.	Statistic	df	Sig.
Rotarod test	1 sham	.158	10	.200*	.947	10	.637
	2 OVX	.156	10	.200*	.907	10	.262
	3 OVX+PNF100	.177	10	.200*	.948	10	.644
	4 OVX+PNF300	.177	10	.200*	.897	10	.201
	5 OVX+E2	.194	10	.200*	.965	10	.842
Inverted screen test	1 sham	.251	10	.073	.912	10	.295
	2 OVX	.189	10	.200*	.898	10	.209
	3 OVX+PNF100	.145	10	.200*	.956	10	.739
	4 OVX+PNF300	.175	10	.200*	.934	10	.487
	5 OVX+E2	.229	10	.145	.916	10	.326

*. This is a lower bound of the true significance.

a. Lilliefors Significance Correction

Table S4. Dunnett's test (Rotarod test and Inverted screen test).

Multiple Comparisons

Dunnett t (2-sided)[a]

Dependent Variable		Std. Error	Sig.	95% Confidence Interval

			Mean Difference (I-J)			Lower Bound	Upper Bound
Rotarod test	2 sham	1 OVX	49.80000*	6.62490	.000	33.0304	66.5696
	3 PNF100	1 OVX	15.90000	6.62490	.068	-.8696	32.6696
	4 PNF300	1 OVX	33.50000*	6.62490	.000	16.7304	50.2696
	5 E2	1 OVX	24.60000*	6.62490	.002	7.8304	41.3696
Inverted screen test	2 sham	1 OVX	43.10000*	7.74031	.000	23.5069	62.6931
	3 PNF100	1 OVX	15.30000	7.74031	.165	-4.2931	34.8931
	4 PNF300	1 OVX	26.40000*	7.74031	.005	6.8069	45.9931
	5 E2	1 OVX	21.40000*	7.74031	.028	1.8069	40.9931

*. The mean difference is significant at the 0.05 level.

a. Dunnett t-tests treat one group as a control, and compare all other groups against it.

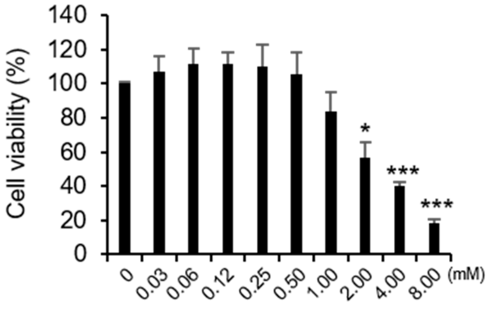

Figure S1. Effects of PNF on the viability of primary differentiated myoblast cells treated with the indicated concentrations for 72 h. (n=3). *p < 0.05, **p < 0.01, and ***p < 0.001.

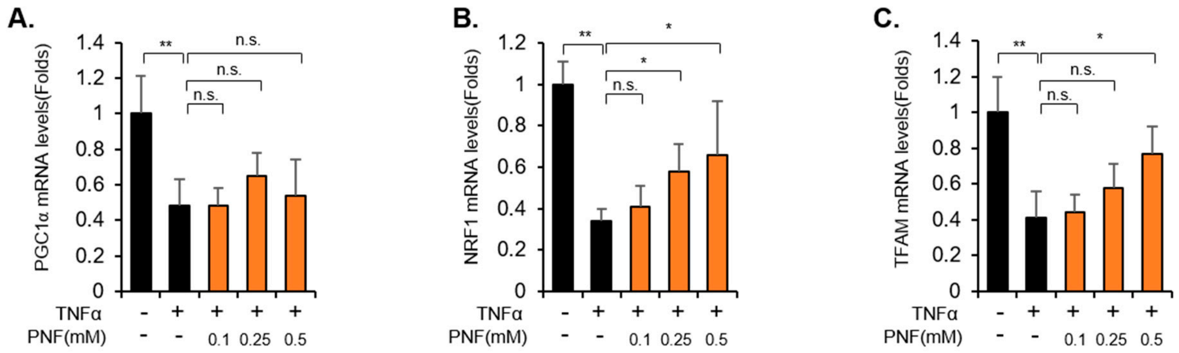

Figure S2. PNF selectively recovers the TNFα-induced reduction of NRF1 and TFAM transcripts, but not PGC1α in C2C12 cells. TNFα (10 ng/ml), PNF was treated in cells for 24 hours. (A) PGC1a, (B) NRF1, (C) TFAM. *p < 0.05, **p < 0.01, ***p < 0.001. N.S., not significant.
